# Pathway Analysis for Genome-Wide Association Study of Basal Cell Carcinoma of the Skin

**DOI:** 10.1371/journal.pone.0022760

**Published:** 2011-07-28

**Authors:** Mingfeng Zhang, Liming Liang, Mousheng Xu, Abrar A. Qureshi, Jiali Han

**Affiliations:** 1 Department of Dermatology, Clinical Research Program, Brigham and Women's Hospital, Harvard Medical School, Boston, Massachusetts, United States of America; 2 Department of Epidemiology and Biostatistics, Cancer Center, Nanjing Medical University, Nanjing, China; 3 Department of Biostatistics, Harvard School of Public Health, Boston, Massachusetts, United States of America; 4 Department of Epidemiology, Harvard School of Public Health, Boston, Massachusetts, United States of America; 5 Channing Laboratory, Department of Medicine, Brigham and Women's Hospital, Harvard Medical School, Boston, Massachusetts, United States of America; Ohio State University Medical Center, United States of America

## Abstract

**Background:**

Recently, a pathway-based approach has been developed to evaluate the cumulative contribution of the functionally related genes for genome-wide association studies (GWASs), which may help utilize GWAS data to a greater extent.

**Methods:**

In this study, we applied this approach for the GWAS of basal cell carcinoma (BCC) of the skin. We first conducted the BCC GWAS among 1,797 BCC cases and 5,197 controls in Caucasians with 740,760 genotyped SNPs. 115,688 SNPs were grouped into gene transcripts within 20 kb in distance and then into 174 Kyoto Encyclopedia of Genes and Genomes pathways, 205 BioCarta pathways, as well as two positive control gene sets (pigmentation gene set and BCC risk gene set). The association of each pathway with BCC risk was evaluated using the weighted Kolmogorov-Smirnov test. One thousand permutations were conducted to assess the significance.

**Results:**

Both of the positive control gene sets reached pathway p-values<0.05. Four other pathways were also significantly associated with BCC risk: the heparan sulfate biosynthesis pathway (p  =  0.007, false discovery rate, FDR  =  0.35), the mCalpain pathway (p  =  0.002, FDR  =  0.12), the Rho cell motility signaling pathway (p  =  0.011, FDR  =  0.30), and the nitric oxide pathway (p  =  0.022, FDR  =  0.42).

**Conclusion:**

We identified four pathways associated with BCC risk, which may offer new insights into the etiology of BCC upon further validation, and this approach may help identify potential biological pathways that might be missed by the standard GWAS approach.

## Introduction

Recently, genome-wide association studies (GWASs) have often been conducted to investigate the genetic susceptibility to complex diseases, including skin cancer [1]. Cancer of the skin (including both melanoma and non-melanoma skin cancers) is the most common form of cancer, accounting for more than 50% of all cancers. Basal cell carcinoma (BCC) accounts for approximately 80 percent of all non-melanoma skin cancers, and its incidence is increasing [2]. Although GWASs have identified several new *loci* associated with BCC [3], [4], the most significant SNPs that fulfilled a stringent statistical “genome-wide” significance criterion often account for only a small proportion of genetic susceptibility. More attention should be paid to the rest of the genetic information, which may offer a deeper understanding of the genetics of complex diseases [5]. Combining the modest association signals in the GWAS data with information on biological pathways and networks, the emerging pathway-based approaches are designed to utilize the GWAS data to a greater extent and are likely to yield new insights into disease etiology [6].

It has been suggested that multiple susceptibility genes for a single disease tend to have some related functions or aggregate in certain pathways [7]. For example, some genes associated with BCC risk are related to pigmentation [1]. The pathway-based approach, which evaluates the cumulative contribution of the functionally related genes, may help uncover the associations of genes with small effect sizes that cluster within common biological pathways [6]. Using an approach based on the gene-set enrichment analysis algorithm [8], pathway analyses have been applied to the GWASs of several complex diseases, and some novel candidate disease-susceptibility pathways have been revealed [9], [10], [11], [12].

Here we report on the results of a pathway analysis on GWAS of BCC to identify the biological pathways associated with BCC risk, which may provide new insights into the etiology of BCC.

## Materials and Methods

### *BCC GWAS*

#### Ethics Statement

This study was approved by the Human Research Committee at the Brigham and Women's Hospital (Boston, MA) with written informed consent from all participants.

#### Description of Study Populations

The BCC GWAS comprised 1,797 BCC cases and 5,197 controls, which were from four pre-existing GWAS sets [13], [14]. The description of BCC GWAS dataset was presented in a manuscript currently in press [15]. Briefly, the four component GWAS sets reflect the type 2 diabetes case-control study nested within the Nurses' Health Study (T2D_NHS, BCC cases = 665, BCC controls = 2,162), the type 2 diabetes case-control study nested within the Health Professionals Follow-up Study (T2D_HPFS, BCC cases = 597, BCC controls = 1,555), the coronary heart disease case-control study nested within the NHS (CHD_NHS, BCC cases = 253, BCC controls = 765), and the coronary heart disease case-control study nested within the HPFS (CHD_HPFS, BCC cases = 282, BCC controls = 715). All the cases were self-reported BCC patients, and we excluded those who had diagnosis of other common cancers before diagnosis of BCC based on common cancers reported by the National Cancer Institute and American Cancer Society. For BCC controls, we also excluded those individuals who had diagnosis of BCC or other common cancers. Those common cancers included melanoma, SCC, breast cancer, endometrial cancer, ovarian cancer, colorectal cancer, bladder cancer, lung cancer, pancreatic cancer, kidney (renal cell) carcinoma, leukemia, Non-Hodgkin Lymphoma, thyroid cancer, and oral cancer. Overlapping individuals among the four GWAS sets were removed. All the individuals in our study are of European ancestry and live in the United States. The study protocol was approved by the Institutional Review Boards of Brigham and Women's Hospital and the Harvard School of Public Health. Detailed descriptions of the study populations for each study are presented in the Methods S1.

#### Genotyping and Quality Control

We used the genotype data from four pre-existing GWAS sets (T2D_NHS, T2D_HPFS, CHD_NHS, and CHD_HPFS) [13]. The genotyping was previously conducted using the Affymetrix 6.0 array and the information on genotyping and quality control was previously described [13]. For each study, the detailed information was summarized in the Methods S1.

#### Association analysis

Associations between each SNP and BCC risk were determined using a multivariable logistic regression model; Age and the first three principal components of genetic variation (Methods S1) were adjusted in the model within each of the four sets. The case-control status of T2D and CHD was also adjusted in the sensitivity analysis. For each SNP, within-cohort association results were combined in an inverse variance–weighted meta-analysis using METAL software [16].

### Pathway data construction

We collected pathway data from the Kyoto Encyclopedia of Genes and Genomes (KEGG) and BioCarta databases; 174 KEGG pathways and 205 BioCarta pathways that contain at least 10 but at most 200 genes represented by qualified SNPs were tested. The cutoff of 10–200 genes was selected as a good balance for gene sets to reduce the multiple-testing issue and to avoid testing overly narrow or broad functional categories. We also designed two positive control gene sets, the pigmentation gene set and BCC risk gene set, to test this approach. We selected the genes based on information gathered by the previous GWAS studies on the pigmentation traits and BCC risk. The pigmentation gene set included *SLC45A2*, *IRF4*, *EXOC2*, *TYPR1*, *TPCN2*, *TYR*, *KITLG*, *SLC24A4*, *OCA2*, *MC1R*, *ASIP*, *HERC2*; and the BCC risk gene set included *PADI6*, *RHOU*, *SLC45A2*, *TERT*, *CLPTM1L*, *KLF14*, *CDKN2A*, *CDKN2B*, *TYR*, *KRT5*, *MC1R*, *ASIP*, *PTCH1*.

### Statistical Method of the Pathway Analysis

We applied the method of Wang et al. [11] to conduct the pathway-based GWAS analysis. Specifically, we first calculated the statistic value for the association of each SNP with BCC risk. Then, we associated the SNPs located within 20 kb upstream or downstream of gene transcript with each gene, and assigned the highest statistical value among all the SNPs located in this region as representative of this gene. All the genes were assigned to pathways as previously defined. Then the association of each pathway with BCC (denoted by Enrichment Score, ES) was evaluated using the weighted Kolmogorov-Smirnov-like running sum statistic, which reflects the overrepresentation of genes within this pathway at the top of the entire ranked list of genes in the genome. We used a permutation procedure (1,000 permutations, permuting the case-control status and re-calculating the pathway-based ES) to assess the significance of the statistical value. A normalized enrichment score was calculated for each pathway to allow direct comparison of pathways of different sizes. The false discovery rate (FDR) was calculated to keep the proportion of expected false positive findings below a certain threshold [17]. We set the significance level for the pathway analysis as p value<0.05 and FDR<0.5. The detailed algorithm of pathway analysis was described by Wang et al [11].

## Results

Basic characteristics of the 1,797 BCC cases and 5,197 controls in our study are presented in Table S1. As shown in the table, the BCC cases were more likely to have red or blonde hair and lifetime severe sunburns that blistered, and they tended to have poorer ability to tan than the controls (Table S1).

Of the 740,760 genotyped SNPs, 366,550 SNPs located within 20 kb upstream or downstream of gene transcripts were assigned to the genes. Among them, 115,688 SNPs were ultimately assigned to 4,891 genes within the pre-defined pathways (107,521 SNPs to 4,538 genes in the KEGG pathways and 34,301 SNPs to 1,298 genes in the BioCarta pathways). A total of 174 KEGG pathways and 205 BioCarta pathways that contained between 10 and 200 eligible genes were used for the pathway analysis.

Both of the positive control gene sets reached a nominal p-value<0.05 (p  =  0.001 for the pigmentation gene set and p  =  0.03 for the BCC risk gene set). As shown in Table S2, 10 out of 12 genes in the pigmentation gene set had representative SNPs that reached p value<0.05 for the association with BCC (range 0.02 to 3.4E-7, and the two that did not reach significance had p values of 0.05 and 0.09), and seven out of 13 genes in the BCC risk gene set had SNPs with p value<0.05 (range 0.03 to 3.4E-7, and the remaining six had p values from 0.11 to 0.16).

Besides the two positive control gene sets, 10 out of the 174 KEGG pathways and eight out of the 205 BioCarta pathways reached a significance level of p<0.05, and four also reached a FDR of less than 0.5. Those were the heparan sulfate biosynthesis pathway (p  =  0.007, FDR  =  0.35), the mCalpain pathway (p  =  0.002, FDR  =  0.12), the Rho cell motility signaling pathway (p  =  0.011, FDR  =  0.30), and the nitric oxide pathway (p  =  0.022, FDR  =  0.42, Table 1). Complete association results for all the pathways in the KEGG database are listed in Table S3 (a), and those in the BioCarta database are listed and Table S3 (b)).

**Table 1:** Pathways with significant enrichment in the BCC GWAS.

Database	Pathway	Gene Count^1^	FDR^2^	Pathway Enrichment p-value	Genes with *P_BCC_*<0.01^3^	SNP^4^	SNP *P_BCC_*
KEGG	Heparan sulfate biosynthesis pathway	19	0.35	0.007	HS3ST3A1	rs8074072	1.93E-03
					HS6ST2	rs7881124	2.37E-03
					HS6ST3	rs8002510	3.43E-03
					HS6ST1	rs11890277	5.43E-03
					HS3ST1	rs1024034	6.45E-03
					EXTL3	rs12544882	9.74E-03
					EXT2	rs7950395	9.75E-03
BioCarta	mCalpain pathway	24	0.18	0.002	ITGA1	rs1833556	2.69E-04
					MYLK	rs820455	8.99E-04
					EGF	rs6824594	9.55E-04
					CAPNS2	rs4784549	6.13E-03
					PXN	rs11611541	7.07E-03
					PRKACG	rs1590944	8.31E-03
					ITGB1	rs7079624	8.66E-03
BioCarta	Rho cell motility signalling pathway	32	0.30	0.011	MYLK	rs820455	8.99E-04
					ROCK1	rs1481280	9.06E-04
					BAIAP2	rs9903763	1.44E-03
					OPHN1	rs5965550	3.70E-03
					PIP5K1B	rs963707	4.37E-03
					ACTR3	rs2279839	4.84E-03
					ARHGEF1	rs11665965	5.40E-03
BioCarta	Nitric oxide pathway	30	0.42	0.022	VEGFA	rs13211073	4.54E-04
					RYR2	rs2805434	9.35E-04
					PRKG1	rs10762543	1.92E-03
					BDKRB2	rs874284	4.73E-03
					NOS3	rs3763486	7.79E-03
					CHRM1	rs4592425	8.24E-03
					PRKACG	rs1590944	8.31E-03
					TNNI1	rs6697016	8.70E-03

1 The number of the genes with SNPs tested in BCC GWAS;

2 Based on 1000 permutations, with 174 tested pathways for KEGG darabase and 205 tested pathways for Biocarta darabase;

3 *P_BCC_* is p-value of SNP for BCC association. Only the genes whose representative SNPs reached *P_BCC_*<0.01 are listed here and the information of all enriched genes in the four pathways are listed in Table S3;

4 Representative SNP for each gene with the smallest *P_BCC_*.

Thirteen out of 19 genes in the heparan sulfate biosynthesis pathway had SNPs with p-value for marginal effect on BCC risk (P_BCC_)<0.05; the same was true for 14 out of 24 genes in the mCalpain pathway, 16 out of 32 genes in the Rho cell motility signaling pathway, and 16 out of 30 genes in the nitric oxide pathway. The representative SNP of each gene in the four pathways that had P_BCC_<0.01 are listed in Table 1, and the representative SNPs of all the genes in these pathways are listed in Table S4.

According to the pathway definition in the BioCarta database, there are some overlaps between the mCalpain pathway (24 genes) and the other two pathways: 3 genes (*MYLK, MYL2 and TLN1*) in the Rho cell motility signaling pathway (32 genes); and 6 genes (*PRKACG, PRKACB, PRKAR2B, PRKAR1A, PRKAR1B, PRKAR2A*) in the nitric oxide pathway (30 genes). After excluding the 3 genes shared between the mCalpain pathway and the Rho cell motility signaling pathway, the association obviously attenuated for both pathways (p  =  0.04 and FDR  =  0.61 for the mCalpain pathway, and p  =  0.05 and FDR  =  0.84 for the Rho cell motility signaling pathway). The association remained similar after excluding the 6 genes shared between the mCalpain pathway (p  =  0.003 and FDR  =  0.12) and the nitric oxide pathway (p  =  0.04 and FDR  =  0.76). No other overlap was found among the remaining pathways. In addition, based on the concern that the case-control status of T2D and CHD may introduce potential bias to the associations between the SNPs and BCC risk, a sensitivity analysis was conducted by adjusting for the case-control status. As a result, no substantial change was detected for the associations before and after the adjustment.

## Discussion

Genome-wide association studies primarily focus on marginal effects for individual markers. Given that some of the susceptibility genes for a given disease share related functions, a pathway-based approach that evaluates the cumulative contribution of the functionally related genes can utilize GWAS data to a greater extent. This method has recently shown its potential strength in application to GWASs in several complex diseases [9], [10], [11], [12]. Here we report the results of a pathway analysis to investigate the GWAS associations for BCC. Gene-sets that contained pigmentation genes or BCC risk genes identified from previous GWASs were significantly associated with BCC in our pathway analysis, which suggests that the pathway-based approach can effectively incorporate the modest effects of genes with related functions. In addition, we identified four other pathways significantly associated with BCC risk as defined by the KEGG and BioCarta databases: the heparan sulfate biosynthesis pathway, the mCalpain pathway, the Rho cell motility signaling pathway, and the nitric oxide pathway.

The mCalpain pathway ranked first among the 205 BioCarta pathways tested in this study (p  =  0.002 and FDR  =  0.18). Calpain is a class of Ca^2+^-dependent cysteine proteases with two major ubiquitously expressed isoforms: μ-calpain (or calpain I) and m-calpain (or calpain II). One of the most carefully studied functions of the calpains is the regulation of integrin-mediated cell migration [18]. mCalpain is believed to be membrane bound and functions at the trailing edge of the migrating cell to cleave the integrins in response to growth factor receptor signals [18], [19], which may play a role in the invasion of tumor cells into surrounding stroma during the development of BCC. In addition to cell migration, a number of protein substrates of calpains have been identified as playing very important roles in cell proliferation, apoptosis, and cell cycle control [20], [21], [22], [23], [24]. The assessed m-calpain expression level appeared to be inversely correlated to apoptotic activity in various cell types [20], [25], and the over-expression of m-calpain has been detected in human cancer [26]. To date, calpain inhibitors have been used widely to trigger apoptosis in various cancer cell lines [27]. However, as for BCC, only one study has reported that the expression of u-calpain in BCCs was markedly elevated compared to normal human skin, and no difference was found for m-calpain [28]. In our study, although we detected only a weak association between the m-calpain polymorphisms and BCC risk (representative p  =  0.05), a stronger association was identified for the m-calpain pathway, which suggests that m-calpain has its effects through interaction with other genes in the pathway.

The other two pathways associated with BCC risk in the BioCarta database were the Rho cell motility signaling pathway and the nitric oxide pathway. After excluding the genes shared with the mCalpain pathway, the association for the Rho cell motility signaling pathway became non-significant, suggesting that the association was driven by the genes shared with the mCalpain pathway. There is evidence from previous study that calpain mediates integrin-induced signaling at a point upstream of Rho family members [29]. It is certainly plausible that BCC tumorigenesis requires the mCalpain pathway for local invasion into the epidermis and through the basement membrane zone into the dermis. Another plausible explanation is an integrin-mediated immune response that requires adhesion of circulating anti-tumor T cells to the dermal endothelial cells followed by migration of T cells into tissues – an aberration of a T cell response to BCC may allow the tumor to grow unabated in skin.

The association of the nitric oxide pathway remained significant after excluding the genes shared with the mCalpain pathway, which suggests an independent effect. Nitric oxide (NO) is a signaling molecule that plays a critical role in vasodilatation, neurotransmission and immunity, and has been shown to be cytostatic or cytotoxic for cancer cells [30]. NO is also produced in the skin and its associated microvasculature, and has complex implications in skin homeostasis and diseases [31]. The NO pathway in the BioCarta database focuses mainly on the sub-network of NOS3. NOS3, also named as endothelial cell constitutive NOS or eNOS, plays an important role in the regulation of vascular endothelial function [32], and the expression of NOS3 was reported to be down-regulated in basal cell carcinomas when compared with normal skin [33]. A significant association was also detected between *NOS3* polymorphisms and BCC risk in our study (representative p  =  0.01). A characteristic clinical feature of BCCs is a translucent papule or nodule with prominent telangiectasia, presumably an angiogenic response propagated by the growing skin cancer. A NOS-predominant BCC may potentially be able to attract a risk blood supply for continued growth and NOS (via NOS inhibitors) may be considered a therapeutic target.

As for the KEGG pathways, the heparan sulfate biosynthesis pathway was significantly associated with BCC risk (p  =  0.007 and FDR  =  0.35). Heparan sulfate (HS) is a glycosaminoglycan covalently attached to serine residues in a proteoglycan core protein. Heparan sulfate proteoglycans (HSPGs) have been known to play vital roles in every step of tumor progression: allowing cancer cells to proliferate, escape from immune response, invade neighboring tissues, and metastasize to distant sites [34]. Several cancers including melanoma have shown aberrant modulation of several key HS biosynthetic enzymes [35], [36], [37], [38]. However, no study has been reported on BCC, and our finding suggests the potential importance of this pathway in the etiology of BCC, relevant in the case of nodular or morpheaform BCCs where dermal invasion is commonly seen.

There are some limitations to our approach. First, current knowledge on human gene functions is incomplete, and the pre-defined pathways cannot comprehensively represent the functionally related genes in the human genome. We used the KEGG and BioCarta pathways in this study, which represent relatively well-defined known biological pathways. To minimize multiple-testing concerns, we did not include some other pathway databases with more broad functional categories such as the Gene Ontology (GO) [39], [40]. Secondly, the assignment of SNPs to genes in physical proximity may not always be accurate. A gene may be regulated in *trans* by genetic variants that are physically distant from the structural gene [41]. Third, given the modest sample size, the FDRs of the identified pathways are not particularly low. Further replication using additional samples and functional investigation would be required to illustrate the true effect behind these pathways.

In conclusion, we applied a pathway-based approach to further explore the GWAS data of BCC and identified several pathways associated with BCC risk, which was missed in the traditional GWAS. These findings may suggest new insights into the etiology of BCC upon further replication. Functional studies are also warranted to characterize the biological functions of these pathways in BCC development.

## Supporting Information

Table S1 **Characteristics of BCC cases and controls in this study.**
(XLS)

Table S2 **The representative SNP information for the two positive control pathways.**
(XLS)

Table S3 **Association results for the 174 KEGG pathways and 205 BioCarta pathways.** (a) Association results for the 174 KEGG pathways; (b) Association results for the 205 BioCarta pathways.
(XLS)

Table S4 **The representative SNP information for the four pathways with significant enrichment in the BCC GWAS.**
(XLS)

Methods S1 **Description of the GWAS on BCC.**
(DOC)
