# Supplementary material for: Pathway Analysis for Genome-Wide Association Study of Basal Cell Carcinoma of the Skin
Source: PLoS One. 2011 Jul 28;6(7):e22760. doi: 10.1371/journal.pone.0022760 (PMC3145747; doi:10.1371/journal.pone.0022760)
Supplement: Methods S1 — Description of the GWAS on BCC. (DOC) [file pone.0022760.s005.doc]

**Methods S1**

**Description of the GWAS on BCC**

***Description of Study Populations***

**Nurses’ Health Study (NHS):** The NHS was established in 1976, when 121,700 female U.S. registered nurses between the ages of 30 and 55, residing in 11 larger U.S. states, completed and returned an initial self-administered questionnaire on their medical histories and baseline health-related exposures, forming the basis for the NHS cohort. Biennial questionnaires with collection of exposure information on risk factors have been collected prospectively. Overall, follow-up has been very high; after more than 20 years approximately 90% of participants continue to complete questionnaires. From May 1989 through September 1990, we collected blood samples from 32,826 participants in the NHS cohort.

**Health Professionals Follow-up Study (HPFS):** In 1986, 51,529 men from all 50 U.S. states in health professions (dentists, pharmacists, optometrists, osteopath physicians, podiatrists, and veterinarians) aged 40-75 answered a detailed mailed questionnaire, forming the basis of the study. The average follow-up rate for this cohort over 10 years is >90%. Between 1993 and 1994, 18,159 study participants provided blood samples by overnight courier.

**The four GWAS sets included in the BCC GWAS:** Detailed descriptions for four GWAS sets were published previously [1,2].

**1) Type 2 diabetes (T2D) case-control study nested within the NHS and HPFS:** Diabetes cases were defined as self-reported incident diabetes confirmed by a validated supplementary questionnaire in the NHS and HPFS. The diagnosis of T2D was made using the National Diabetes Data Group (NDDG) proposed criteria before 1998, and using the American Diabetes Association’s diagnostic criteria during the 1998 and 2000 cycles. The non-diabetic control subjects were matched to cases on age, month and year of blood draw, and fasting status [1].

**2) Coronary heart disease (CHD)** **case-control study nested within the NHS and HPFS:** In both the NHS and HPFS, participants who had reported an incident CHD event on the follow-up questionnaire were contacted for confirmation and permission to review medical records was requested. Medical records for deceased participants were also sought for deaths that were identified by families and postal officials and through the National Death Index. Physicians blinded to the participant’s questionnaire reports reviewed all medical records. Fatal CHD cases were identified primarily through review of medical records, as previously described [2]. Among participants who provided blood samples and who were without cardiovascular disease or cancer at blood draw, incident CHD cases occurring after blood draw were selected as cases. Controls were selected, in a 2:1 ratio matched to cases on age, smoking, and month of blood return.

***Laboratory Assays and Quality Control (QC) procedures***

The genotyping was previously performed in the four GWAS sets (T2D_NHS, T2D_HPFS, CHD_NHS, and CHD_HPFS) by using the Affymetrix 6.0 array [1]. Detailed QC procedures were provided as follows.

**1) T2D_NHS and T2D_HPFS:** The NHS and HPFS T2D GWA scans are a component of the Gene Environment-Association Studies (GENEVA) under the NIH Genes, Environment and Health Initiative (GEI). The QC procedures were previously described in details [1]. Briefly, genotypic data first passed Broad's initial QC which included SNP fingerprints for sample tracking and early detection of sample misidentification, missing call rates of ≥5%, the use of a HapMap control to check genotype quality independent of study samples and tracking of reagent and instrumental performance. Of the 909,622 SNP probes on the array, 879,071 passed the Broad's technical QC standards for 3,473 NHS samples, and 874,517 SNP probes passed this QC stage for 2,640 HPFS samples.

Genotype data were subsequently released for further QC to the GENEVA Coordinating Center at the University of Washington. Relatedness was evaluated using pairwise identity-by descent estimation using 80k SNPs in a method of moments approach implemented in PLINK software [3]. In NHS, five pairs of duplicate samples were identified and removed. Gender was confirmed by examining the mean of the intensities of SNP probes on the X and Y chromosomes. One male sample was mis-identified as a female sample and was excluded. Twenty-seven subjects with highly variable intensity data, determined by analyzing relative intensity (‘Log-RRatio’) and allelic imbalance (‘BAlleleFreq’, BAF) [4], and 22 samples having a missing call rate of ≥2% were also removed. In HPFS, four pairs of duplicate samples were excluded. Six samples with evidence of contamination and 20 with highly variable intensity data were excluded; as were 13 with missing call rates ≥2%. Population structure was investigated by principal component analysis [5]. Unrelated genetically inferred European ancestral women and men passing QC were included in the study, which were 3286 NHS samples and 2486 HPFS samples for the final analysis. As for SNPs, we applied the same QC parameters to both scans: excluding SNPs which were monomorphic, had a missing call rate of ≥2%, more than one discordance, significant deviations from Hardy-Weinburg equilibrium (HWE, *P* < 1 × 10^−4^) and an MAF of < 0.02. Duplicate SNPs (assayed with different probes) were also removed. A total of 704,409 SNPs for NHS samples and 706,040 SNPs for HPFS samples passed QC. We further excluded 59 SNPs with MAF < 0.01 or HWE *P* < 1 × 10^−6^ among the BCC controls in NHS and 72 SNPs in HPFS. Finally, we included 704,350 SNPs for NHS samples and 705,968 SNPs for HPFS in our study.

**2) CHD_NHS and CHD_HPFS:** QC procedures for CHD_NHS and CHD_HPFS were similar to those for the T2D sets. Briefly, PLINK software [3] was used for data cleaning. The data were cleaned separately for NHS and HPFS according to thresholds recommended by the GENEVA consortia described above. 907,370 SNPs passed the technical QC for 1,157 NHS samples and 1,330 HPFS samples. We excluded one pair of duplicate samples, one individual with gender-misidentification and eight non-Caucasians in the NHS set, as well as one individual with gender-misidentification and 16 non-Caucasians in the HPFS set. 1,146 NHS samples and 1,313 HPFS samples remained in the final analysis. As for the SNPs, we excluded those met any of following criteria: 1) MAF <0.02; 2) call rate <95%; 3) P for HWE < 1 × 10^−4^ in control groups; 4) concordance rate <95% among the duplicated QC samples; 5) significant difference in missing rates between cases and controls (P < 1 × 10^−5^). After applying the QC filter, 721,316 SNPs remained for NHS and 724,881 for HPFS. We further excluded 31 SNPs with MAF of < 0.01 or HWE *P* < 1 × 10^−6^ among the BCC controls in NHS and 26,657 SNPs in HPFS. Finally, we included 721,285 SNPs remained for NHS and 698,224 for HPFS in our study.

***Principal Components of Genetic Variation***

The principal components were calculated for all individuals on the basis of ca. 10,000 unlinked markers using the EIGENSTRAT software [6]. We adjusted for the 3 largest principal components of genetic variation in the unconditional logistic regression model of each GWAS set.

**References:**

1. Qi L, Cornelis MC, Kraft P, Stanya KJ, Linda Kao WH, et al. (2010) Genetic variants at 2q24 are associated with susceptibility to type 2 diabetes. Hum Mol Genet 19: 2706-2715.

2. Rimm EB, Giovannucci EL, Willett WC, Colditz GA, Ascherio A, et al. (1991) Prospective study of alcohol consumption and risk of coronary disease in men. Lancet 338: 464-468.

3. Purcell S, Neale B, Todd-Brown K, Thomas L, Ferreira MA, et al. (2007) PLINK: a tool set for whole-genome association and population-based linkage analyses. Am J Hum Genet 81: 559-575.

4. Peiffer DA, Le JM, Steemers FJ, Chang W, Jenniges T, et al. (2006) High-resolution genomic profiling of chromosomal aberrations using Infinium whole-genome genotyping. Genome Res 16: 1136-1148.

5. Patterson N, Price AL, Reich D (2006) Population structure and eigenanalysis. PLoS Genet 2: e190.

6. Price AL, Patterson NJ, Plenge RM, Weinblatt ME, Shadick NA, et al. (2006) Principal components analysis corrects for stratification in genome-wide association studies. Nat Genet 38: 904-909.
